# The Development of Diabetes and Diabetic Ketoacidosis Following Immunotherapy Treatment: A Systematic Review of Case Reports

**DOI:** 10.7759/cureus.57894

**Published:** 2024-04-09

**Authors:** Stephanie Nagy, Michelle Demory Beckler, Atif Hussein, Marc M Kesselman

**Affiliations:** 1 Rheumatology, Dr. Kiran C. Patel College of Osteopathic Medicine, Nova Southeastern University, Fort Lauderdale, USA; 2 Microbiology and Immunology, Dr. Kiran C. Patel College of Allopathic Medicine, Nova Southeastern University, Fort Lauderdale, USA; 3 Hematology and Oncology, Memorial Cancer Institute, Pembroke Pines, USA

**Keywords:** checkpoint inhibitors, continuous glucose monitoring, diabetic keto acidosis, diabetes, cancer immunotherapy

## Abstract

As cancer continues to be the leading cause of death worldwide, additional therapeutic options other than traditional platinum-based chemotherapy have become available that target tumor cells in innovative ways. Immunotherapies (e.g., immune checkpoint inhibitors (ICI)) ramp up the immune system to target cancer cells, providing patients with more personalized and tumor cell-specific treatment options. This new age oncological treatment option has been found to provide a more meaningful and stronger alternative to traditional chemotherapy, resulting in longer periods of remission and milder side effects. However, because ICI heightens the immune system, resultant autoimmune conditions can occur. One of the most recently shown adverse effects of ICI are extreme hyperglycemia (i.e., type 1 diabetes) and diabetic ketoacidosis (DKA). To determine the incidence of immunotherapy-induced diabetes, a systematic literature review was performed using CINHAL, EBSCO, MEDLINE, and Web of Science. A total of 403 articles were initially screened, with a final 28 case reports included. The results show that checkpoint inhibitors were found to be most commonly associated with new-onset diabetes as opposed to traditional chemotherapy. Additionally, 41% of patients developed autoimmune diabetes and DKA after being placed on a single therapy of pembrolizumab (targets PD-1: programmed cell death protein 1). However, the pathological process underlying the development of endocrinopathies after treatment with ICI continues to be under investigation.

## Introduction and background

Over the last decade, there have been significant developments in cancer treatment modalities besides traditional chemotherapy, which was developed in the 1960s. Platinum-based chemotherapies (i.e., Cisplatin, Carboplatin, and Oxaliplatin) are clinically approved worldwide for cancer treatment and function by inducing DNA platination leading to apoptosis [1]. Traditional chemotherapy drugs have strong therapeutic effects on tumor cells; however, due to their non-specific nature, they can lead to systemic toxicity and significant side effects of nephrotoxicity, neurotoxicity, ototoxicity, and myelosuppression [1]. These undesirable and life-threatening side effects have led to the evolution of immunotherapy that harnesses and amplifies the immune system. By increasing activation or decreasing deactivation of the immune system, a patient’s immune cells are exploited to target cancer cells, while also sparing non-cancer cells. Cancer continues to be the leading cause of death worldwide, resulting in 9958133 million deaths and over 19292789 new cancer cases in 2020, as per the most recent report by the World Health Organization [2]. As cancer rates soar, immunotherapy has become an increasingly prevalent treatment option as monotherapy or adjuvant therapy to chemotherapy.

Traditional chemotherapy targets DNA, RNA, or mRNA to inhibit cell proliferation or activate apoptosis, thereby, limiting tumor growth; however, chemotherapy is unable to differentiate between rapidly dividing cancer and normal healthy cells [3]. As a result, patients can develop the side effects of myelosuppression, mucositis, nausea, vomiting, diarrhea, alopecia, fatigue, sterility, infertility, and immunosuppression [3]. Immunotherapy, on the other hand, can provide a more tailored and precise treatment option to target cancer cells, while limiting these significant side effects [4]. Additionally, it appears that immunotherapies tend to lead to higher survival rates compared to traditional treatments [5]. Patients receiving traditional chemotherapy were found to have a diminished progression-free period compared to immunotherapy of 6.9 months and 11 months, respectively. However, it was found that patients receiving a combination of chemotherapy and immunotherapy had the longest progression-free survival rate of 11.8 months [5].

Immunotherapy aims to utilize or modulate the patient’s immune system to specifically and rapidly target cancer cells [4]. One of the largest classes of immunotherapy to treat a wide spectrum of cancers is monoclonal antibody immunotherapy. A subclass of monoclonal antibodies utilizes tumor-specific antigens to produce cancer-specific in vitro antibodies. These antibodies are then introduced into the patient and specifically target cancer-specific antigens. This type of monoclonal antibody therapy has become a key method of immunotherapy because not only does it specifically target tumor antigens but it also provides long-term anti-tumor responses [6]. A subtype of monoclonal antibody therapy includes anti-cytotoxic T-lymphocyte-associated antigen-4 (CTLA-4) antibodies and programmed cell death protein 1 pathway/programmed cell death protein 1 ligand (PD-1/PDL-1)-specific antibodies. Both of these antibody types downregulate the deactivation of T-cells, leading to an increase in the total number of activated T-cells [7]. By inducing a loss of T-cell deactivation, these antibodies promote T-cell anti-tumor activity [4].

Chimeric antigen receptor therapy (CAR-T) exploits a patient’s T-cells to specifically target cancer antigens. The patient's T-cells are collected and reprogrammed in vitro to display CAR on their surface. These receptors are specific for tumor antigens. Subsequently, the genetically engineered CAR-T cells are reintroduced into the patient and are specific for tumor cells [8]. CAR-T therapy and monoclonal antibody therapy provide rapid advances in precision medical treatments.

Furthermore, natural killer (NK) cells are used as a form of immunotherapy in cancer treatment. NK cells are a key feature of the innate immune system, with cytotoxic and cytokine effects. As such, NK cells are activated without antibody or T-cell production, therefore making them powerful in the elimination of cancer cells through their recognition of absent major histocompatibility (MHC) class I on tumor cells [9].

Immunotherapy has opened the doors for new modes of oncological treatment options and has been crucial in providing more personalized and precise cancer-targeting therapies; however, as with any treatment, the benefits are not without adverse effects. Patients receiving immunotherapy may develop fatigue, skin rash, pneumonitis, colitis, diarrhea, arthritis, hepatitis, thyroiditis, pancreatitis, and hypophysitis [10]. Studies have shown the development of endocrinopathies in 4% to 30% of patients [11]. The mechanism behind the autoactivation is yet to be well understood; it is postulated that it may be due to the activation of autoreactive CD8+ T-cells against antigens within the beta-cell including proinsulin and pre-proinsulin antigen, tyrosine phosphatase-like insulinoma antigen, islet-specific glucose-6-phosphate protein, glutamic acid decarboxylase-65, zinc transporter protein 8, and islet amyloid polypeptide [12].

Thyroid dysfunction and hypophysitis are the most prevalent adverse effects within the endocrine system [10]. Damage to the pancreas is a rare event; however, when present, it may present with life-threatening adverse effects. Large fluctuations of thyroid and pancreatic hormone levels have recently been shown in patients to unexpectedly develop diabetes or a life-threatening diabetic ketoacidosis (DKA) crisis [10].

This systematic case review aims to analyze reported cases of the development of diabetes and DKA after non-diabetic oncological patients receive immunotherapy treatment and evaluate the importance of implementing continuous glucose monitoring (CGM) for all patients receiving immunotherapy.

## Review

Methods

Search Strategy

A systematic literature review was performed using CINHAL, EBSCO, MEDLINE, and Web of Science. The search was conducted using the Boolean operators “AND” and “OR” between the keywords selected for the literature search as follows, “Immunotherapy AND Diabetes OR Diabetic Ketoacidosis.” The articles were filtered to be within the last decade, 2013-2023, in the English language, and relevance was evaluated in a hierarchical approach that considered the title, abstract, and then the full manuscript. For articles that were not freely accessible, the Nova Southeastern University library database was used to gain access.

Selection Criteria

**Figure 1:**
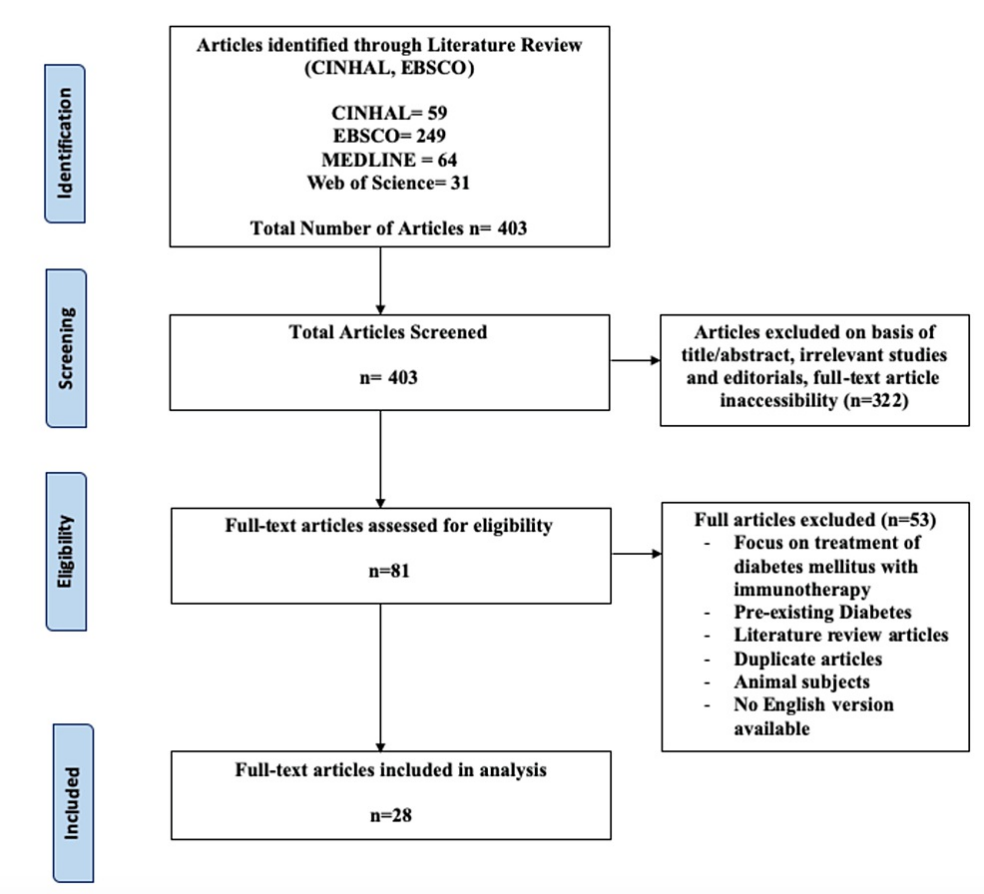
PRISMA diagram PRISMA: preferred reporting items for systematic reviews and meta-analyses

The included studies were considered eligible if they discussed reported cases of patients not previously diagnosed with diabetes, developing diabetes, or DKA following immunotherapy treatment. Eligible study designs included patient case reviews. Inclusion criteria included free full-text versions, articles in the English language, and the inclusion of keywords within the title and abstract. Exclusion criteria included study designs, which were secondary analysis-based studies, literature reviews, non-in vivo human studies, inaccessibility of the full-text version, duplicate studies if the patient was previously diagnosed with diabetes, and if the study had no mention of the development of diabetes or DKA after immunotherapy. The Joanna Briggs Institute critical appraisal checklists were utilized to assess the attributes of each article to determine their efficacy and reliability [13]. The preferred reporting items for systematic reviews and meta-analyses (PRISMA) were used to develop a flow diagram of the selection criteria (Figure 1).

Results

A total of 403 articles were identified based on the keyword searches of the four databases (CINHAL, EBSCO, MEDLINE, and Web of Science). After screening and determining the eligibility of the articles, 403 articles were included. A total of 322 articles were excluded based on their title/abstract, inaccessibility of the full-text version, or if the studies were irrelevant to the established keywords or editorial. Out of the 81 remaining, 53 were removed based on the exclusion criteria of non-human participants, inaccessible full-text articles, duplicate studies, and literature reviews or if the focus of the paper was on the treatment of diabetes with immunotherapy or if the patients had pre-existing immunotherapy. Twenty-eight case studies were analyzed with 29 patients as Luo et al. investigated two patient cases within the same case report [14].

**Figure 2:**
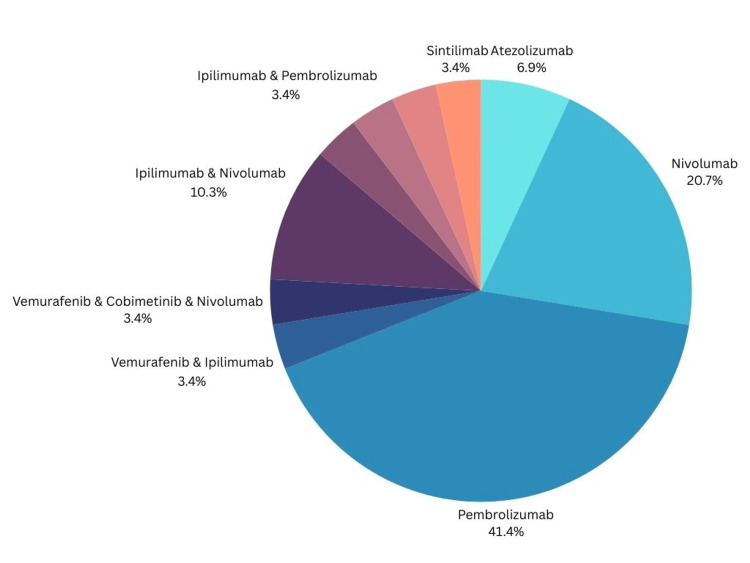
Percentage distribution of immunotherapy medications received by patients

**Figure 3:**
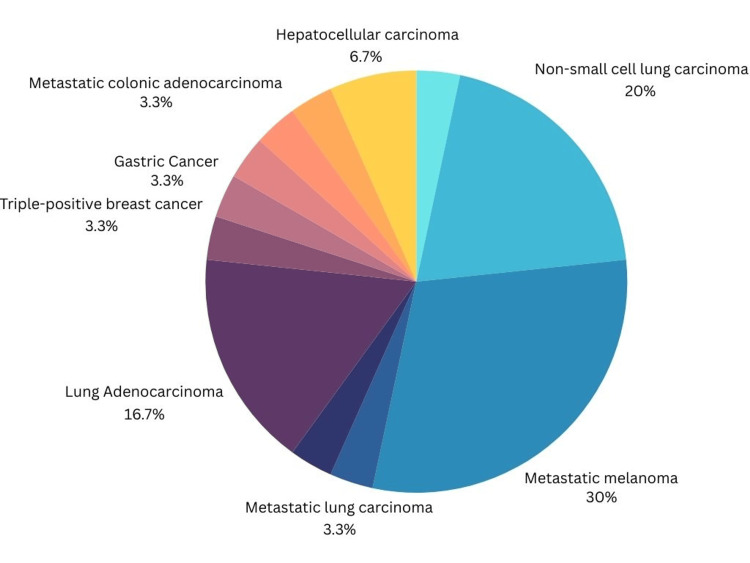
Distribution of oncological diagnosis for immunotherapy patients

Table 1 depicts the case studies included in the analysis including publication year, number of subjects, type of immunotherapy received, mechanism of action (MOA) of the immunotherapy, and the pertinent patient findings from each case. Figure 2 indicates the breakdown of the immunotherapies received by patients. The most common immunotherapy received leading to diabetes or DKA was found to be single therapy with pembrolizumab at 41% followed by single therapy with nivolumab at 21% (Figure 2). Patients received immunotherapy for a variety of oncological diagnoses including metastatic urothelial cancer, non-small cell lung carcinoma, metastatic melanoma, metastatic lung carcinoma, small cell lung cancer, lung adenocarcinoma, triple-positive breast cancer, gastric cancer, metastatic colonic adenocarcinoma, esophageal carcinoma, nasopharyngeal carcinoma, and hepatocellular carcinoma. Patients diagnosed with metastatic melanoma were found to develop diabetes or DKA more commonly followed by non-small cell lung cancer and lung adenocarcinomas, as seen in Figure 3.

**Table 1:** Summary of patient case reports MOA: mechanism of action; ICI: immune checkpoint inhibitors; PD-L1: programmed death-ligand 1; CTLA-4: cytotoxic T-lymphocyte-associated antigen-4; MEK: mitogen-activated protein kinase; DKA: diabetic ketoacidosis; HbA1C: glycated hemoglobin

Article	Author	Year	No. of Patients	Type of Cancer	Immunotherapy	MOA	Summary of Pertinent Findings
Anti-PD-L1 Atezolizumab-induced autoimmune diabetes: a case report and review of the literature	Hickmott et al. [15]	2017	1	Metastatic urothelial cancer	atezolizumab	PD-1 inhibitor	A 57-year-old man with recurrent metastatic urothelial cancer was placed in a clinical trial for atezolizumab. By the fifth cycle of treatment, the patient developed polydipsia, weight loss, and fatigue. The patient’s blood work showed plasma glucose levels of 24 mmol/L (432 mg/dL), ketones of 6.3 mmol/L, elevated anion gap of 19.1, bicarbonate of 16.9 mmol/L, venous pH of 7.3, and a Hb1Ac of 7.5%. The patient was diagnosed with autoimmune diabetes secondary to a PD-L1 inhibitor and treated with insulin and intravenous fluids.
Diabetes mellitus secondary to treatment with ICI	Venetsanaki et al. [16]	2019	1	Non-small cell lung carcinoma	nivolumab	PD-1 inhibitor	A 71-year-old patient with non-small cell lung carcinoma was treated with nivolumab. After 10 months of treatment, the patient presented with generalized weakness, polyuria, and polydipsia with plasma glucose of 471 mg/dL (26.2 mmol/L), HbA1C of 7.8%, and no ketone bodies. The patient was diagnosed with nivolumab-associated T1DM and treated with insulin and intravenous fluids.
Pembrolizumab: a case of drug-induced autoimmune diabetes mellitus and colitis	Singh [17]	2018	1	Metastatic melanoma	pembrolizumab	PD-1 inhibitor	A 70-year patient with metastatic melanoma experienced diarrhea, vomiting, mucositis, and loss of appetite following the third treatment with pembrolizumab. The lab results showed a blood glucose of 51 mmol/L (918 mg/dL), pH of 6.99, ketonemia of 6.7 mmol/L, and lactate of 3.8 mmol/L. The patient was managed in the ICU with fluid therapy and IV insulin. The patient was discharged on insulin glargine and insulin aspart.
Pembrolizumab-induced auto-immune type-1 diabetes in a patient with metastatic melanoma	Reslan et al. [18]	2018	1	Metastatic melanoma	pembrolizumab	PD-1 inhibitor	A 79-year-old patient with metastatic melanoma was treated with pembrolizumab. After the fifth cycle of the treatment, the patient presented two months later with a plasma glucose of 39.8 mmol/L (716.4 mg/dL) and Hb1Ac of 7.5%. The patient was diagnosed with PD-1 inhibitor autoimmune diabetes mellitus and managed with insulin.
A case of pembrolizumab‑induced type‑1 diabetes mellitus and discussion of immune checkpoint inhibitor‑induced type 1 diabetes	Chae et al. [19]	2016	1	Metastatic lung carcinoma	pembrolizumab	PD-1 inhibitor	A 76-year-old patient with metastatic lung cancer was treated with pembrolizumab. After the second cycle was found to have elevated glucose levels without evidence of polyuria and polydipsia, the patient was diagnosed with ICI‑induced type 1 diabetes that was corrected with insulin and the patient continued with their next cycle of pembrolizumab.
A case report of insulin-dependent diabetes as immune-related toxicity of pembrolizumab: presentation, management and outcome	Hansen et al. [20]	2016	1	Metastatic melanoma	Vemurafenib and ipilimumab	BRAF inhibitor and CTLA-4 Inhibitor	A 58-year-old patient diagnosed with metastatic melanoma was treated with vemurafenib and ipilimumab, for 16 months resulting in a stable disease. Two years following treatment, the patient developed new intra-abdominal, subcutaneous, and liver metastasis and was started on PD-1 therapy with pembrolizumab. After receiving 17 cycles of therapy, they presented with a plasma glucose of 408 mg/dL (22.7 mmol/L) and elevated HbA1c of 9.7% resulting in the diagnosis of insulin-dependent diabetes. The patient was treated with insulin, and blood glucose levels neutralized.
Autoimmune diabetes induced by PD-1 inhibitor-retrospective analysis and pathogenesis: a case report and literature review	Gauci et al. [21]	2017	1	Metastatic melanoma	Vemurafenib, cobimetinib, and nivolumab	BRAF inhibitor, MEK inhibitor, and PD-1 inhibitor	A 73-year-old patient diagnosed with metastatic melanoma was treated with vemurafenib and cobimetinib initially but with the progression of disease a PD-1 inhibitor nivolumab was added. After three cycles, the patient developed abdominal pain, vomiting, asthenia, polyuria, and polydipsia. The lab tests showed glycemia of 27.78 mmol/L (500 mg/dL), ketonuria, glucosuria, bicarbonate of 18 mmol/L, and Hb1Ac of 8.8%. The patient was diagnosed with type 1 diabetes with acute renal failure and treated with insulin. The patient then began therapy on nivolumab without any episode of hyperglycemia.
Diabetic ketoacidosis: an adverse reaction to immunotherapy	Keerty et al. [22]	2020	1	Small cell lung cancer	Ipilimumab and nivolumab	CTLA-4 inhibitor and PD-1 inhibitor	A 49-year-old female was treated with nivolumab and ipilimumab for small cell lung cancer. The patient received two doses and following the second dose presented to the emergency room with intermittent and cramping abdominal pain and mild respiratory distress. Blood tests showed elevated plasma glucose of 384 mg/dL (21.3 mmol/L), decreased bicarbonate levels of 14 mmol/L, and HbA1c of 6.6%. Urinalysis was positive for ketones and glucosuria. The patient was treated with a bolus of saline, regular insulin, and an insulin drip.
A severe case of diabetic ketoacidosis and new-onset type 1 diabetes mellitus associated with anti-glutamic acid decarboxylase antibodies following immunotherapy with pembrolizumab	Kedzior et al. [23]	2021	1	Lung adenocarcinoma triple-positive breast cancer	pembrolizumab	PD-1 inhibitor	A 51-year-old female was treated with chemotherapy and pembrolizumab for lung adenocarcinoma and triple-positive breast cancer. They presented to the emergency department two weeks after their second dose of pembrolizumab with abdominal pain, dizziness, diarrhea, and vomiting. Testing indicated elevated glucose above 1123 mg/dL (62.4 mmol/L), elevated ketones, an anion gap of 24 mmol/L, and a pH of 6.94. The patient was diagnosed with DKA and cared for in the ICU with standard insulin therapy and rehydration.
Diabetic ketoacidosis as a delayed immune-related event after discontinuation of nivolumab	Mae et al. [24]	2021	1	Gastric cancer	nivolumab	PD-1 inhibitor	A 59-year-old man received treatment for gastric cancer with nivolumab for 12 cycles over nine months. After discontinuation of the medication, four months later the patient presented to the emergency department with an elevated HbA1c of 10.6%, elevated blood glucose of 690 mg/dL (38.3 mmol/L), elevated anion gap of 15.2 mmol/L, weight loss, excessive thirst, and excessive urination. Urinalysis displayed ketone in urine and glucosuria. The patient was diagnosed with DKA and treated with lactated ringer rehydration therapy, bolus of insulin, and continuous insulin therapy.
Pembrolizumab-induced diabetes mellitus presenting as diabetic ketoacidosis in a patient with metastatic colonic adenocarcinoma	Kichloo et al. [25]	2020	1	Metastatic colonic adenocarcinoma	pembrolizumab	PD-1 inhibitor	A 77-year-old female was treated for her metastatic colonic adenocarcinoma with pembrolizumab. The patient received three cycles of the medications prior to presenting to the emergency room, four weeks after their last dose. Blood results showed a bicarbonate level of 11 mmol/L, an elevated anion gap of 24 mmol/L, HbA1c of 8.8%, and fasting glucose of 747 mg/dL (41.5 mmol/L). Urinalysis presented glycosuria and ketones. The patient was diagnosed with DKA and treated in ICU with rehydration and insulin therapy. They were discharged on basal insulin.
Diabetic ketoacidosis as an immune-related adverse event from pembrolizumab in non–small cell lung cancer	Leonardi et al. [26]	2017	1	Non-small cell lung cancer	pembrolizumab	PD-1 inhibitor	A 66-year-old male with non-small cell lung cancer presented with fatigue, polyuria, and polydipsia following the third dose of pembrolizumab. Blood tests showed elevated anion gap, ketonemia, and ketonuria. The patient was treated with insulin IV therapy and discharged on insulin aspart and subcutaneous insulin glargine. The patient continued their pembrolizumab therapy.
Combined checkpoint inhibitor therapy causing diabetic ketoacidosis in metastatic melanoma	Changizzadeh et al. [27]	2017	1	Metastatic melanoma	Nivolumab and Ipilimumab	PD-1 inhibitor and CTLA-4 inhibitor	A 42-year-old male with metastatic melanoma treated with nivolumab and ipilimumab for three cycles presented to the emergency room with nausea, vomiting, and diarrhea. Blood tests showed serum glucose to be elevated at 728 mg/dL (40.4 mmol/L), bicarbonate of 14 mmol/L, and an elevated anion gap of 25 mmol/L. The patient was treated with intravenous insulin bolus and drip with IV fluids. The patient was diagnosed with DKA.
Nivolumab-induced autoimmune diabetes mellitus presenting as diabetic ketoacidosis in a patient with metastatic lung cancer	Godwin et al. [28]	2017	1	Non-small cell lung cancer	nivolumab	PD-1 inhibitor	A 24-year-old woman was treated with nivolumab for non-small cell lung cancer; two weeks after her second dose, she presented to the emergency department with abdominal pain, nausea, and weakness. Blood tests showed a glucose of 738 mg/dL (41 mmol/L), blood pH of 7.12, anion gap elevation of 30 mmol/L, and urine ketones above 80 mg/dL. She was treated in the ICU with IV fluids and insulin IV.
Pembrolizumab- and ipilimumab-induced diabetic ketoacidosis and isolated adrenocorticotropic hormone deficiency: a case report	Porntharukchareon et al. [29]	2020	1	Non-small cell lung cancer	pembrolizumab and ipilimumab	PD-1 inhibitor and CTLA-4 inhibitor	A 70-year-old man with stage 4B non-small lung cancer with pleural and liver metastases treated with pembrolizumab and ipilimumab presented with fatigue, nausea, and vomiting to the emergency department. The blood tests showed an elevated blood glucose at 794 mg/dL (44.1 mmol/L), ketones at 6.3 mmol/L, bicarbonate at 13 mmol/L, and an elevated anion gap at 24 mmol/L. The patient received IV hydration and IV insulin therapy.
Pembrolizumab and diabetes: a case of diabetic ketoacidosis in a patient with metastatic melanoma	Lolomari et al. [30]	2023	1	Metastatic melanoma	pembrolizumab	PD-1 inhibitor	A 75-year-old male was treated with pembrolizumab for metastatic melanoma. They received three cycles of pembrolizumab; after the first cycle, they experienced joint aching and itchiness; after the second, they experienced chest tightness and decreased exercise tolerance, and 10 weeks after the third dose, they began to experience nausea, polyuria, polydipsia, and weight loss. The patient's blood test relieved an elevated blood glucose of 26 mmol/L (468 mg/dL), pH of 7.2, and blood ketones of 4 mmol/L, and they were diagnosed with DKA. The patient was treated in hospital with insulin with no complications.
A case of pembrolizumab-induced diabetic ketoacidosis and hyperthyroidism in a patient with recurrent esophageal adenocarcinoma	Salangsang et al. [31]	2023	1	Esophageal carcinoma	pembrolizumab	PD-1 inhibitor	A 53-year-old male treated with pembrolizumab for esophageal carcinoma presented to the emergency department with shortness of breath, dry mouth, polyuria, and polydipsia. Blood results showed an elevation of blood glucose at 451 mg/dL (25.1 mmol/L), HbA1c at 7.2%, blood gas pH of 7.06 mmol/L, bicarbonate og 5 mmol/L, and a urinalysis positive for glucose and ketones. The patient was diagnosed with DKA and treated with IV hydration and insulin and recovered without complication.
Nivolumab-induced fulminant diabetic ketoacidosis followed by thyroiditis	Tzoulis et al. [32]	2018	1	Lung adenocarcinoma	nivolumab	PD-1 inhibitor	A 56-year-old women presented to the emergency department after their third cycle of nivolumab for metastatic lung adenocarcinoma. The patient was diagnosed with DKA with the lab results of glucose of 47 mmol/L (846 mg/dL), ketones of 7.5 mmol/L, pH of 6.95, bicarbonate of 6.6 mmol/L, and Hb1Ac of 8.2%. The patient was treated with IV saline and continuous insulin infusion.
Nivolumab-induced type 1 diabetes mellitus as an immune-related adverse event	Miyoshi et al. [33]	2016	1	Metastatic melanoma	nivolumab	PD-1 inhibitor	A 66-year-old female received nivolumab for metastatic melanoma with vaginal and cervical metastases. After six doses, they presented with nausea, vomiting, and weight loss. Blood tests showed a glucose of 531 mg/dL (29.5 mmol/L), HbA1c of 7.3%, pH of 7.29, bicarbonate levels of 12.2 mmol/L, and high anion gap of 23.8 mmol/L. The patient was diagnosed with DKA, received isotonic saline and continuous insulin infusion, and was discharged on a subcutaneous insulin regime.
Type 1 diabetes mellitus induced by PD-1 inhibitors in China: a report of two cases	Luo et al. [14]	2022	2	Nasopharyngeal carcinoma, non-small cell lung carcinoma	nivolumab and Pembrolizumab	PD-1 inhibitor	A 81-year-old female treated with nivolumab for nasopharyngeal carcinoma received six doses for her treatment. Six months following the final sixth dose, the patient presented to the emergency department with ketonuria, elevated glucose of 1124 mg/dL (62.4 mmol/L), pH of 7.23, bicarbonate of 14.5 mmol/L, and a Hb1Ac of 8.8%. The patient was diagnosed with fulminant type 1 diabetes mellitus and prescribed subcutaneous insulin injects. A 76-year-old patient diagnosed with non-small cell lung cancer received Pembrolizumab after the 11th dose; the patient presented to the emergency department with vomiting, diarrhea, weight loss, lethargy, without polyuria, polydipsia, and polyphagia. The patient’s blood work showed ketonuria, elevated glucose levels of 590.40 mg/dL (32.8 mmol/L), pH of 7.36, bicarbonate levels of 21.50 mmol, and Hb1Ac of 4.6%. They were treated with subcutaneous insulin.
New-onset autoimmune diabetes mellitus presenting as diabetic ketoacidosis in association with pembrolizumab therapy and long term follow-up: case report	Pachpande et al. [34]	2022	1	Lung adenocarcinoma	pembrolizumab	PD-1 inhibitor	A 62-year-old male receiving pembrolizumab and chemotherapy for their lung adenocarcinoma. The patient received 12 cycles of pembrolizumab and two weeks following the last dose reported vomiting, diarrhea, polydipsia, and excessive thirst. Blood work revealed bicarbonate levels of 12 mmol/L, elevated anion gap of 25 mmol/L, glucose of 600 mg/dL (33.3 mmol/L), and a pH of 7.28. The patient was diagnosed with DKA, managed on an IV insulin drip, and discharged on subcutaneous insulin.
Toripalimab-associated diabetes mellitus: a case report from the community of Southern China	Zhang et al. [35]	2023	1	Metastatic melanoma	toripalimab	PD-1 inhibitor	A 20-year-old patient diagnosed with a melanoma that was surgically removed was then treated with toripalimab. After three cycles, the patient developed an elevated blood glucose of 7.3 mmol/L (131.4 mg/dL) and weight loss; at the time they were treated with metformin and gliclazide. Two months later the patient presented with a fasting blood glucose of 20.7 mmol/L (372 mg/dL), Hb1Ac of 10.4%, positive for glycosuria, and no ketonuria. The patient was treated with IV insulin and discharged on subcutaneous injection of insulin aspart and insulin degludec.
Atezolizumab-induced autoimmune diabetes in a patient with metastatic lung cancer	Sothornwit et al. [36]	2019	1	Non-small cell lung cancer	atezolizumab	PD-1 inhibitor	A 52-year-old women with non-small cell lung cancer presented after five cycles of treatment with atezolizumab for their non-small cell lung cancer with DKA. The blood glucose was 332 mg/dL (18.4 mmol/L), Hb1Ac was 7.9%, with an elevated anion gap, bicarbonate was 9.9 mmol/L, anion gap of 24 mmol/L, elevated serum B-hydroxybutyrate of 5.91 mmol/L, and pH of 6.9. They were treated with a bolus of insulin and discharged on subcutaneous insulin aspart and insulin glargine.
Complete pathological response with diabetic ketoacidosis to the combination of sintilimab and anlotinib in an unresectable hepatocellular carcinoma patient: a case report	Fu et al. [37]	2022	1	Hepatocellular carcinoma	Sintilimab and anlotinib	PD-1 inhibitor and tyrosine kinase inhibitor	A 42-year-old man was treated with sinitilimab and anlotinib; following the 11th cycle, they experienced nausea, vomiting, thirst, polyuria, ketonuria, and ketonemia. The blood sugar level was 17.0 mmol/L (306 mg/dL); the patient was diagnosed with DKA. They were placed on insulin glargine, insulin aspart, and regular blood glucose monitoring.
Sintilimab-induced autoimmune diabetes in a patient with the anti-tumor effect of partial regression	Wen et al. [38]	2020	1	Hepatocellular carcinoma	Sintilimab	PD-1 inhibitor	A 56-year-old male was treated with sintilimab for their unresectable hepatocellular carcinoma. After eight cycles of sintilimab, the patient experienced polyuria, polydipsia, elevated fast glucose of 22.2 mmol/L (399.6 mg/dL), HbA1c of 7.8%, pH of 7.27, bicarbonate of 12.9 mmol/L, and lactate level of 1.8 mmol/L. The patient was diagnosed with sintilimab-induced new-onset autoimmune diabetes and treated with rehydrate therapy, electrolyte acid-base balance therapy, and insulin. The patient was discharged on insulin aspart and insulin glargine.
New onset autoimmune diabetes mellitus and hypothyroidism secondary to Pembrolizumab in a patient with metastatic lung cancer	Cunha et al. [39]	2022	1	Lung adenocarcinoma	Pembrolizumab	PD-1 inhibitor	A 59-year-old female with lung adenocarcinoma underwent a right upper lobe lobectomy and was placed on Erlontinib; however, cancer metastasized to the brain and patient was switched to Pembrolizumab. Three weeks after their first dose of pembrolizumab, they experienced symptoms of polyuria, polydipsia, weight loss, and vomiting. Blood tests showed 554 mg/dL (30.8 mmol/L), ketonemia of 4 mmol/L, elevated anion gap of 18.9 mmol/L, pH of 7.31, bicarbonate of 17.7 mmol/L, lactate of 1.5 mmol/L, and HbA1c of 5.6%. The patient was diagnosed with DKA and treated with isotonic fluid replacement, insulin IV infusion, and transitioned to basal-bolus insulin regiment. The patient was discharged on daily insulin glargine and lispro.
Late-onset acute type 1 diabetes mellitus 7 months after discontinuation of Pembrolizumab against lung cancer	Ichihara et al. [40]	2023	1	Lung adenocarcinoma	Pembrolizumab	PD-1 inhibitor	A 68-year-old female was treated for lung adenocarcinoma in the right lobe with mediastinal lymph node swelling. The patient was given carboplatin, pemetrexed, and pembrolizumab; after four cycles, the patient had a good response and was continued on pemetrexed and pembrolizumab. After the seventh cycle, the medications were stopped due to the progression of the disease. 37 months later the patient presents to the emergency department with thirst, polyuria, fatigue, and weight loss. Blood results showed a glucose level of 348 mg/dL (19.2 mmol/L), Hb1Ac of 11.3%, anion gap elevation of 18.2 mmol/L, and bicarbonate of 11.8. Urinalysis showed glycosuria and ketonuria. The patient was diagnosed with DKA. They were treated with insulin therapy, IV hydration, and electrolyte correction.
Fulminant diabetes in a patient with advanced melanoma on nivolumab.	Chokr et al. [41]	2018	1	Metastatic melanoma	Nivolumab and ipilimumab	PD-1 inhibitor and CTLA-4 inhibitor	A 61-year-old male with metastatic melanoma to their chest wall and lungs was treated with nivolumab and ipilimumab. After their third cycle, they experienced maculopapular rash and were treated for high-dose prednisone; however, the patient experienced worsening symptoms of fatigue and nausea. The patient presented to the emergency department with malaise, nausea, vomiting, polyuria, dizziness, tachycardia, and tachypnea, and they were hypotensive. Blood results showed a blood glucose of 1211 mg/dL (67.3 mmol/L), bicarbonate level under 10 mmol/L, elevated anion gap above 31 mmol/L, pH of 7.14, beta-hydroxybutyrate of 13.7 mmol/L, and lactic acid of 2.4 mmol/L. Urinalysis was positive for ketonuria and glycosuria. The patient was diagnosed with DKA and managed in the ICU with IV hydration and insulin therapy. The patient was released on basal-bolus insulin glargine and lispro.

In the case studies reviewed, the distribution of patients receiving each form of medication is as follows: atezolizumab (n=2), nivolumab (n=6), pembrolizumab (n=12), vemurafenib and ipilimumab combination therapy (n=1); vemurafenib, cobimetinib, and nivolumab combination therapy (n=1); ipilimumab and nivolumab combination therapy (n=3); ipilimumab and pembrolizumab combination therapy (n=1); toripalimab (n=1); sintilimab and anlotinib combination therapy (n=1); and sintilimab (n=1). Represented statistically in Figure 2.

The types of oncological diagnoses of each patient included in the study were analyzed for prevalence in Figure 3. Patients were diagnosed with the following: metastatic urothelial cancer (n=1), non-small cell lung carcinoma (n=6), metastatic melanoma (n=9), metastatic lung carcinoma (n=1), small cell lung cancer (n=1), lung adenocarcinoma (n=5), triple-positive breast cancer (n=1), gastric cancer (n=1), metastatic colonic adenocarcinoma (n=1), esophageal carcinoma (n=1), nasopharyngeal carcinoma (n=1), and hepatocellular carcinoma (n=2).

Discussion

Diabetes mellitus occurs as a result of a dysfunction within the pancreatic beta-cells that produce insulin, leading to abnormal carbohydrate metabolism and hyperglycemia. Diabetes can be classified as type 1 and type 2. Type 1 diabetes is an autoimmune disorder leading to the breakdown of the beta-cells and the loss of insulin production, often diagnosed in childhood [42]. Type 2 diabetes is the most common, involving the progressive destruction of beta-cells and the development of insulin resistance within cells [42]. Insulin, an endocrine peptide hormone, binds to target cells to initiate glucose uptake, utilization, and storage in the cell. In the absence of insulin, glucose accumulates in the bloodstream, leading to hyperglycemia [43]. Patients with hyperglycemia often present with polyuria, polydipsia, polyphagia, nocturia, blurry vision, weight loss, glycosuria, and hypovolemia [42]. Diagnostic criteria for diabetes include a random blood glucose of over 200 mg/dL (11.1 mmol/L) and present with symptoms of hyperglycemia, as mentioned previously. Patients must fulfill the criteria of fasting plasma glucose to be above 126 mg/dL (7.0 mmol/L) in the absence of hyperglycemic symptoms, or two-hour plasma glucose above 200 mg/dL (11.1 mmol/L) following an oral glucose tolerance test, or an HbA1c of over 6.5% [42]. DKA rapidly occurs over a 24-hour period seen with symptoms of hyperglycemia and, most commonly, nausea, vomiting, abdominal pain, fruity odor to the breath, dry mucosa, poor skin turgor, tachycardia, hypotension, and shock in severe cases [44]. DKA results from decreased levels of insulin secretions leading to elevated levels of glucose as well as alterations in glucagon, catecholamines, cortisol, and growth factor, resulting in the overproduction of glucose by the liver and impaired utilization of glucose in tissues [45]. Classical lab presentations include glucose levels ranging between 350 and 500 mg/dL (19.4 to 27.8 mmol/L), ketones (acetoacetic acid, beta-hydroxybutyric, and acetone) can accumulate in blood and urine, and metabolic acidosis with an anion gap greater than 20 mEq/L in DKA [44]. DKA is critical to diagnose and treat early as, left untreated, it can lead to diabetic coma and death.

Analyzing the medications each reviewed patient received, the prescription of single-therapy pembrolizumab (n=12) and nivolumab (n=6) led to the highest incidences of diabetic crisis at 41.4% and 20.7%, respectively (Figure 2). pembrolizumab and nivolumab are both monoclonal antibodies targeting the PD-1 checkpoint. Monoclonal antibodies are produced in vitro and have a high affinity for the cancer cell surface antigens. Additionally, the Fc portion of the antibody can activate the complement cascade, antibody-dependent cellular cytotoxicity, and antibody-mediated phagocytosis [46].

The mechanism underlying the connection between immunotherapy and the endocrine system is not fully understood. However, there has been increasing investigation into the potential reasoning behind the endocrine side effects of checkpoint inhibitors such as pembrolizumab and nivolumab. As single-therapy of pembrolizumab and nivolumab cumulatively make up 62.1% of the immunotherapy treatment the reviewed patients received prior to developing life-threatening DKA, the main focus should be investigating the potential MOA behind these agents that lead to the targeting of endocrine organs and the subsequent development of endocrinopathies (Figure 2).

Checkpoint inhibitor-associated diabetes is thought to occur as a result of the autoimmune destruction of beta-pancreatic cells. There are numerous hypotheses behind the MOA leading to the development of diabetes in these patients.

Summary of the Current Working Hypotheses

1. PD-1 receptors on T-cells allow the binding and activation of PD-L1 ligands. The binding of PD-1 and PD-L1 has been found to produce an inhibitory signal that regulates activated and autoreactive T-cells and suppresses the immune system. Therefore, PD-1 inhibitors lead to an activated immune system with higher levels of functioning T-cells. Beta-cells within the pancreas harbor PD-1; with the upregulation of T-cells with the use of PD-1 inhibitors, it is postulated that beta-cells may react maladaptively to the elevated autoreactive T-cells leading to its failure to produce insulin resulting in diabetes [46]. 2. Macrophages residing in the pancreas are a key initiator in the development of diabetes in nonobese mice. Anti-colony stimulating factor-1 receptor (anti-CSF-1R) antibodies can remove diabetes-inducing macrophages to reduce the incidence of diabetes. Checkpoint inhibitors may remove the protective effects of anti-CSF-1R antibodies, leading to a greater risk in the development of diabetes; however, the MOA is poorly understood [46]. 3. Traditionally, pancreatic islet antibodies, most commonly GAD65, zinc transporter 8, insulin, and islet antigen 2, develop against beta-cells leading to a deficient production of insulin. In type I diabetic patients, it has been hypothesized that after receiving immunotherapy, an autoimmune reaction may occur, resulting in the production of anti-islet antibodies leading to the development of diabetes following treatment [47]. Additionally, as helper T-cells play a crucial role in antibody production by beta-cells, the use of PD-1 inhibitors may lead to additional autobody production through elevated levels of T-cells.

As the prevalence of immunotherapy-induced diabetes continues to rise, the American Society of Clinical Oncology has produced clinical guidelines for managing endocrine complications arising from ICI [48]. In the management of diabetes, the American Society of Clinical Oncology states that early diagnosis and treatment are crucial. As a result, the guidelines recommend fasting or random blood glucose and HbA1c to be completed before and after administering ICI. Patients must also be educated on the signs and symptoms of hyperglycemia and ketoacidosis for early recognition. If a patient presents with symptoms, obtaining laboratory evaluation for ketones in blood and urine, anion gap, anti-glutamic acid decarboxylase, anti-islet antibodies, insulin, and C-peptide levels is recommended. Patients who are asymptomatic or have mild symptoms, have fasting glucose above 160 mg/dL (8.9 mmol/L), and show no evidence of ketosis should continue their immunotherapy regimen with continuous close follow-up and lab testing. For patients with moderate symptoms with fasting glucose between 160 and 250 mg/dL (8.9-13.9 mmol/L) and evidence of ketosis, it is recommended that ICI be held until glucose control is obtained; insulin therapy and admission into the hospital are recommended when ketosis or new-onset of T1DM symptoms are present. Patients with severe symptoms including DKA or with glucose levels above 250 mg/dL (13.9 mmol/L) should be held until glucose levels are controlled, insulin therapy must be rapidly initiated with an urgent consultation to endocrinology, and should be admitted for symptom management, especially patients who have progressed to DKA [48].

As clinicians shift their focus from a reactive approach to a preventative approach, our recommendation to overcome the rising incidence of diabetes and DKA during or following ICI is the initiation of CGM for all patients receiving ICI. Placing patients on CGM serves as a preventative measure for patients to be alerted firsthand to major shifts in glucose levels before their rapid decline, which often results in emergency room visits and the development of life-threatening DKA. CGM is a wearable device that tracks glucose levels in short intervals to provide a real-time display of glucose levels, monitors the rate of change of glucose levels, and can alert for impending hypoglycemia or hyperglycemia [49]. CGM has been found to reduce acute diabetes complications in type 1 diabetics by 49% and in type 2 diabetics by 39.4% and also reduced the occurrences of DKA by 56.2% in type 1 diabetics and 52.1% in type 2 diabetics. Hospitalizations for hypoglycemia decreased by 10.8%, and for hyperglycemia, they were reduced by 26.5% [50]. Given the consequences of poor glycemic control as a side effect of ICI, it is important for clinicians to recognize the importance of placing patients on CGM at the start of immunotherapy as standard protocol to ensure fluctuations in glucose levels are caught early while allowing patients to continue to receive the life-saving therapy.

Furthermore, Baricitinib, a Janus kinase inhibitor, traditionally used as immunotherapy treatment in rheumatoid arthritis, alopecia areata, and COVID-19 was recently found to preserve the function of beta-cells in patients with diabetes [51]. More research is required to further understand the mechanism; however, placing patients on Baricitinib as an adjuvant therapy may be an additional protocol for preventing diabetes and DKA in patients receiving ICI, such as pembrolizumab and nivolumab.

The limitations associated with case reports include non-random sampling, resulting in the inability to determine causality claims. Additionally, the information provided for patient’s history and lab values varied between articles.

## Conclusions

With the growing use of immunotherapy in the treatment of cancer, immunotherapy-induced type 1 diabetes and DKA are becoming a prominent health concern. Further investigation is required into the underlying mechanism, specifically within the monoclonal antibody subtype of checkpoint inhibitors, which are the major class leading to autoimmune diabetes. Until the pathological process is well understood, healthcare providers must work to prevent the occurrence of those life-threatening endocrinopathies. Given the rapid development of autoimmune diabetes, screening and early recognition are important to avoid the development of DKA. It is recommended as a standardized protocol for healthcare providers to place all patients receiving ICI on a CGM to ensure close monitoring of glucose levels to allow for early treatment and prevention of autoimmune-induced diabetes and DKA.
